# Impacts of COVID-19 pandemic on urban park visitation: a global analysis

**DOI:** 10.1007/s11676-020-01249-w

**Published:** 2020-11-12

**Authors:** Dehui (Christina) Geng, John Innes, Wanli Wu, Guangyu Wang

**Affiliations:** Faculty of Forestry, University of British Columbia, 2424 Main Mall, Vancouver, BC V6T 1Z4 Canada

**Keywords:** COVID-19, COVID-19 response policies, Parks visitation, Stepwise regression analysis, Urban parks

## Abstract

The COVID-19 pandemic has resulted in over 33 million confirmed cases and over 1 million deaths globally, as of 1 October 2020. During the lockdown and restrictions placed on public activities and gatherings, green spaces have become one of the only sources of resilience amidst the coronavirus pandemic, in part because of their positive effects on psychological, physical and social cohesion and spiritual wellness. This study analyzes the impacts of COVID-19 and government response policies to the pandemic on park visitation at global, regional and national levels and assesses the importance of parks during this global pandemic. The data we collected primarily from Google’s Community Mobility Reports and the Oxford Coronavirus Government Response Tracker. The results for most countries included in the analysis show that park visitation has increased since February 16th, 2020 compared to visitor numbers prior to the COVID-19 pandemic. Restrictions on social gathering, movement, and the closure of workplace and indoor recreational places, are correlated with more visits to parks. Stay-at-home restrictions and government stringency index are negatively associated with park visits at a global scale. Demand from residents for parks and outdoor green spaces has increased since the outbreak began, and highlights the important role and benefits provided by parks, especially urban and community parks, under the COVID-19 pandemic. We provide recommendations for park managers and other decision-makers in terms of park management and planning during health crises, as well as for park design and development. In particular, parks could be utilized during pandemics to increase the physical and mental health and social well-being of individuals.

**Electronic supplementary material:**

The online version of this article (10.1007/s11676-020-01249-w) contains supplementary material, which is available to authorised users.

## Introduction

The COVID-19 has been characterized as a pandemic by the World Health Organization due to the high numbers of confirmed cases and deaths, and has posed an unprecedented health crisis to human beings (Chan et al. 2020; Stier et al. 2020; WHO 2020). As of 1 October 2020, the pandemic has caused over 33 million confirmed cases and over 1 million deaths globally (Johns Hopkins University 2020). Restrictions on the use of public spaces, quarantine and social distancing are key measures implemented to tackle the COVID-19 pandemic and protect public health. Countries across the world have introduced policies such as stay-at home-lockdowns, restrictions on public events, social gatherings and public transport, the closure of schools and workplaces, and public COVID-19 information campaigns (Honey-Roses et al. 2020; Ritchie et al. 2020a). With the use of leisure facilities such as shopping malls, restaurants and recreational places, the cancellation of social activities, and the requirements of self-quarantine and social distancing, all being restricted, parks and green spaces have become increasingly popular and important for public health and social benefits (Twohig-Bennett and Jones 2018).

During the COVID-19 pandemic, quarantine and self-isolation, potential health issues and uncertainty, limited outdoor and social activities, social media exposure with negative news, and other pandemic-driven stressors such as financial problems and food insecurity, have all resulted in negative physiological and psychological effects for people. Symptoms include fatigue, tiredness, insomnia, post-traumatic stress symptoms, anxiety, loneliness, confusion, depression and anger. These have posed a threat to humans’ mental and physical health, as well as social cohesion and resilience (Bo et al. 2020; Brooks et al. 2020; Monson et al. 2017; Gao et al. 2020; Wu et al. 2005; Xiang et al. 2020). During the COVID-19 pandemic, parks and green spaces are receiving renewed attention due to their significant and irreplaceable functions, such as providing places for healthy outdoor recreation (Rice and Pan 2020; Rung et al. 2011; Samuelsson et al. 2020). Urban parks and green spaces have been recognized as green infrastructure that provide and deliver environmental, social, psychological and health functions and ecological services for residents. They also provide people with multiple opportunities in terms of recreation and support people health, community cohesion and city sustainability (Chen et al. 2018; Chiesura 2004; Wolff et al. 2020). As well, under health crisis and global pandemic, parks and green spaces clearly benefit human mental and physical health, as well as social well-being (Holland et al. 2018; Thomsen et al. 2013).

Park visitation has changed during the COVID-19 pandemic, though the nature of this change has varied in different countries around the world. According to Google COVID-19 Community Mobility Reports (Ritchie et al. 2020b), park visitor numbers in most countries initially decreased before eventually increasing, and finally reaching levels equal to or even higher than a baseline taken as the mean value of daily park visitors between January 3rd and February 6th, 2020. Detailed analyses of the drivers contributing to changes in park visitor numbers during the COVID-19 pandemic are limited, especially at a global scale (Rice and Pan 2020). This paper therefore addressed the following research questions:

(1) What factors have affected the changes in park visitor numbers during the pandemic?

(2) What kind of relationship exists between daily park visitor numbers and the daily number of COVID-19 cases, and does this relationship vary under government policies, or according to economic, cultural and social factors?

(3) What are the roles of parks and green spaces during the COVID-19 pandemic? How can parks and green spaces systems best be managed to better serve the needs of urban residents during a pandemic?

## Materials and methods

### Framework for COVID-19 impacts on park visitation analysis

**Fig. 1:**
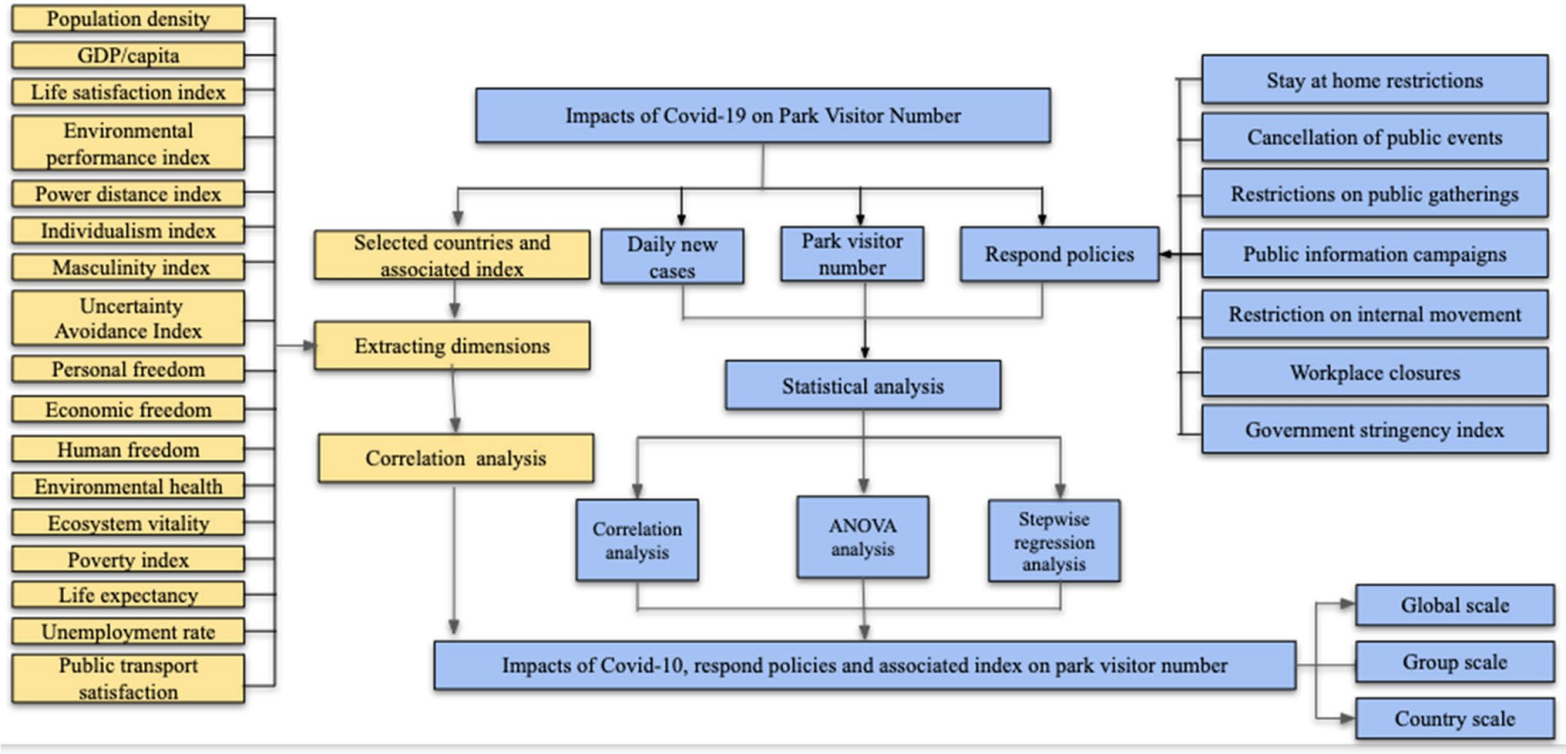
Framework for COVID-19 impacts on park visitation analysis

The overall framework of the research is shown in Fig. 1. This summarizes the data collection, analysis and methodology used here to analyze the impacts of COVID-19 on the change of park visitor numbers. The framework was structured and developed into two parts. The first part (in blue) included three categories of variables, including park visitor number changes, COVID-19 daily new cases, and government policies and restrictions. After the data collection, correlation analysis and stepwise regression analysis were used to analyze the impacts of COVID-19 and associated policies on park visitation at global, regional and national levels. The second part (in yellow) of this framework involved data collection based on selected countries’ and regions’ indices associated with social, economic, cultural and governance values and park visitation. This enabled the relationships between park visitation and various indices to be analyzed. A more detailed methodological description is provided below, as each step of the process is explained.

### The geographical scope of the study

**Fig. 2:**
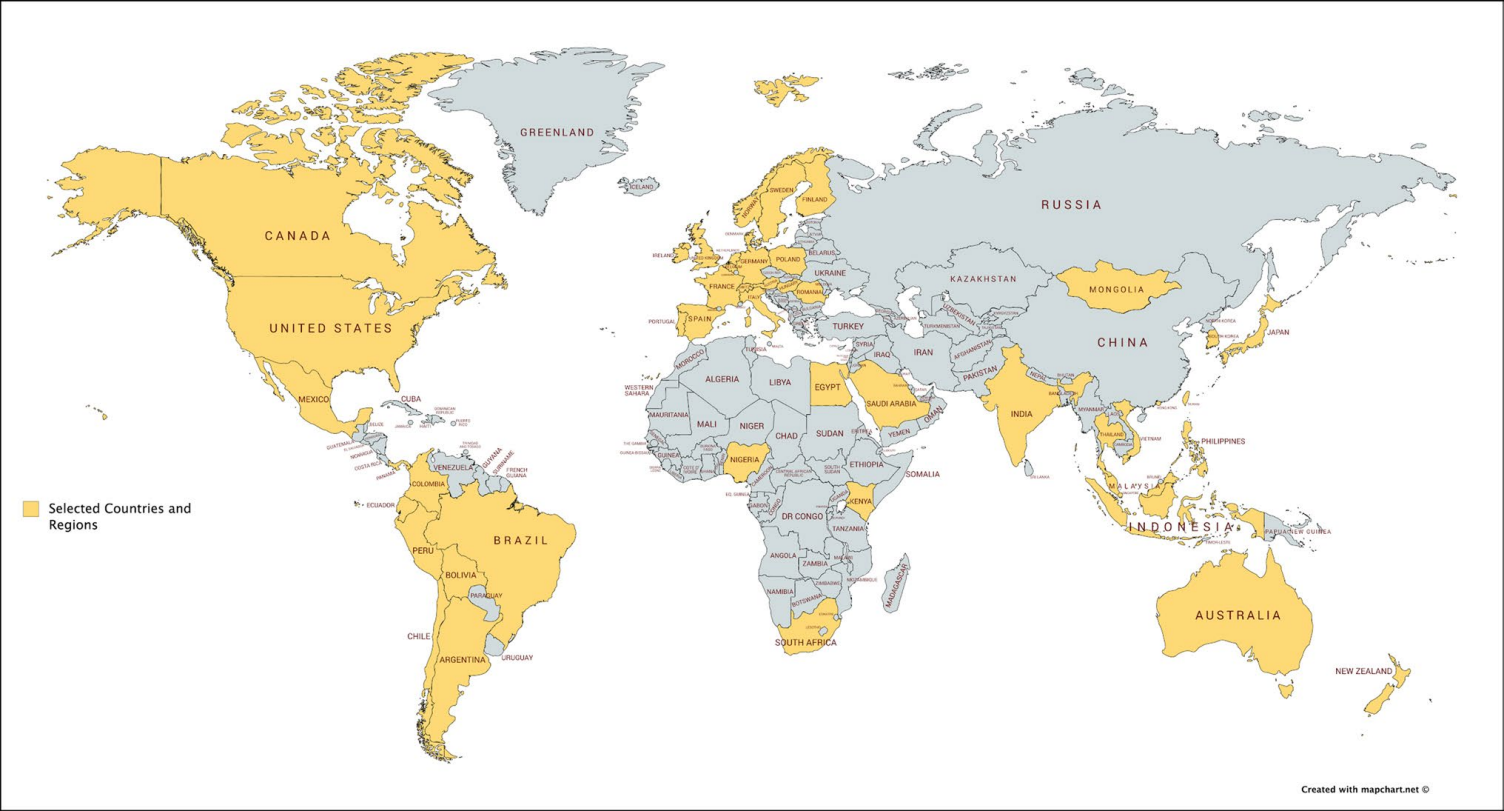
The geographical scope of the study

Countries were selected primarily because they had severe COVID-19 outbreaks. The selection was based on three severity indicators: cumulative number of COVID-19, infection rate and fatality rate. This resulted in the selection of 98 countries. The second step involved assessing data availability, and the last step was to screen and select countries and regions representing diverse economic, social and cultural dimensions. This process led to the selection of 48 countries and regions (in yellow) (Fig. 2).

### Data collection and variable selection

#### Park visitation data during the pandemic

Park visitation data was obtained from the Google Community Mobility Report, which was first released on April 3rd, 2020, and provides a dataset that is regularly updated by Google and shows how the movements of people have changed every day since February 16th, 2020. The dependent variable for this study is the change in the number of park visitors in relation to a Pre-COVID baseline, which is extracted from this dataset. The dataset includes mobility information for individuals, and compares park visitation and visitors’ lengths of stays to the baseline visitor number that was collected in January before the COVID-19 pandemic widely started in 130 countries and regions around the world (Ritchie et al. 2020b). In this dataset, parks include outdoor places like local and community parks, national parks, public beaches, marinas, dog parks, plazas, and public gardens. In this study, park visitation data were statistically extracted from February 16th, 2020 to May 26th, 2020 that covers the first wave of COVID-19 pandemic and right before the Black-Lives-Matter protests were occurred globally.

#### The daily confirmed cases of COVID-19

Data for the number of daily confirmed cases of COVID-19 were obtained from the World Health Organization (WHO) Health Emergency Dashboard. According to the dashboard, the data are refreshed every 15 min, and are accurate at time of refreshing. Another dataset for daily confirmed cases of COVID-19 was used as an additional reference to correct and increase the accuracy of the data, which is published online by Our World in Data. These data were retrieved from the statistics and research work of many different people and organizations. To be consistent with the data for park visitation, the temporal scale of the daily confirmed cases of COVID-19 collection were collected from February 16th, 2020 to May 26th, 2020.

#### Government policies at different stages

Government policies and restrictions at different stages were collected and adapted from the Coronavirus Government Response Tracker (OxCGRT). OxCGRT is a data source published and updated by over 100 researchers at the Blavatnik School of Government at the University of Oxford. The tracker collects information on government responses to COVID-19 from every part of the world. OxCGRT collects and represents available information on 17 indicators of government responses and policies since January 2020 (Ritchie et al. 2020a). Seven of these indices were chosen as indicators: stay-at-home restrictions, cancellation of public events, restrictions on social gathering, public information campaigns, restrictions on internal movement, workplace closure, and the government stringency index. The categories and descriptions of each response are represented in Table S1. Government response data used in this study cover the period February 16th, 2020 to May 26th, 2020.

#### Variables associated with social, economic, cultural and governance aspects

Seventeen indices were collected to represent the economic, cultural, social and government dimensions of each country or region. They were: population density, GDP/capita, life satisfaction index, environmental performance index, power distance index, individualism index, masculinity index, uncertainty avoidance index, personal freedom index, economic freedom index, human freedom index, environmental health index, ecosystem vitality index, poverty index, life expectancy, unemployment rate, and public transport satisfaction. Specific information about each is given in Table S2.

### Statistical analysis

Statistical analyses of the impacts of COVID-19 on park visitation were undertaken at global, regional and national scales, and included correlation analysis, ANOVA analysis and stepwise regression analysis. Stepwise regression analysis was a preferred tool as it involves no previous theoretical knowledge about the predicted impacts of COVID-19 and government responses on park visitation on a global scale. Stepwise regression analysis allows independent variables to be entered into the model based on statistical criteria, such as the *t*-value (Field 2009; Pallant 2010). Independent variables that have significant impacts on park visitation can thus be easier to identify.

Firstly, the impacts on the park visitation of COVID-19 and the subsequent government responses across 48 countries and regions were analyzed. After the global-level analysis, 48 countries and regions were classified into 6 groups based on the correlation between park visitor numbers, daily COVID-19 case increases and government responses. After the classification, stepwise regression analysis was conducted on each group, respectively. In the final step, 1–2 countries or regions were selected in each group as representative and were the subject of more detailed analyses of the drivers and impacts of selected independent variables on changes in park visitor numbers. Finally, a correlation analysis was conducted to investigate the relationships between the 17 indices and changes in park visits.

## Results

### Global-scale analysis of the impacts of COVID-19 on park visitation

Table 1 shows the correlations between the changes in park visitor numbers, COVID-19 daily case increases and the nine government responses for 48 countries. All ten independent variables were significantly correlated with changes in park visitor numbers at a 99% confidence interval. In the global-scale analysis, all variables were negatively correlated with changes in park visits. Among ten independent variables, stay at home restrictions were the most significant factor negatively correlated with changes in the number of park visits, followed by the restrictions on public transport and internal movement. The daily increase in the number of COVID-19 cases had the least significant effect on changes in park visitor numbers in the global-scale analysis.

**Table 1:** Correlation and significance tests between park visitor numbers, COVID-19 and government responses at a global scale

		Daily case increase	Restrictions on stay at home	Public event cancellation	Social gathering restriction	Public information campaign	Internal movement restrictions	Workplace closure	Government stringency index
Park visitor number	Pearson correlation	− 0.052^**^	− 0.511^**^	− 0.346^**^	− 0.341^**^	− 0.141^**^	− 0.395^**^	− 0.405^**^	− 0.461^**^
	Sig. (2-tailed)	0	0	0	0	0	0	0	0
	N	4848	4848	4848	4848	4848	4848	4848	4848

* indicates *P* ≤ 0.05; ** indicates *P* ≤ 0.01, bold font indicates statistical significance

Before applying the stepwise regression analysis, several assumptions were examined. First, the variables were tested for normality, using skewness and kurtosis values to determine whether they fitted a normal distribution with a statistical significance level of 95%. After the significance test, the variance inflation factor (VIF) and tolerance values were calculated to ensure there were no multicollinearity problems. The VIF of all variables were more than 0.1, and the tolerance values were less than 10, indicating that the variables passed the multicollinearity test. The results of the stepwise regression analysis are shown in Tables 2 and 3.

**Table 2:** Variables entered in the stepwise regression model for the global scale

Model	Variable entered	*R*	*R*^2^	Adjusted *R*^2^	Std. Error of the Estimate	R Square Chang	Change Statistics			
							F Change	df1	df2	Sig. F Change
1	Stay at home restriction	0.511	0.261	0.261	36.46459	0.261	1712.843	1	4846	0
2	Daily COVID-19 increase case	0.515	0.266	0.265	36.35891	0.004	29.211	1	4845	0
3	Government stringency index	0.518	0.269	0.268	36.282	0.003	21.564	1	4844	0
4	Social gathering restriction	0.524	0.275	0.274	36.13209	0.006	41.277	1	4843	0
5	Public information campaign	0.531	0.282	0.282	35.95051	0.007	50.047	1	4842	0
6	Public event cancelation	0.534	0.285	0.284	35.89664	0.002	15.542	1	4841	0
7	Workplace closure	0.535	0.286	0.285	35.866	0.001	9.276	1	4840	0.002
8	Internal Movement restrictions	0.535	0.287	0.286	35.85393	0.001	4.259	1	4839	0.039

**Table 3:** Country and region classification based on the correlation between park visitor numbers and COVID-19 daily increase in cases

Significant negative correlation								Significant positive correlation	Pearson correlation	No significant correlation	Pearson correlation
Very strong (0.8 − 1)	Pearson correlation	Strong (0.60– 0.79)	Pearson correlation	Moderate (0.40– 0.59)	Pearson correlation	Weak (0.20–0.39)	Pearson correlation				
Italy	− 0.815^**^	Austria	− 0.655^**^	Argentina	− 0.521^**^	Australia	− 0.251^*^	Denmark	0.397^**^	Canada	− 0.004
Singapore	− 0.880^**^	France	− 0.614^**^	Belgium	− 0.464^**^	Bolivia	− 0.373^**^	Finland	0.365^**^	Ecuador	− 0.187
		Mexico	− 0.613^**^	Colombia	− 0.419^**^	Brazil	− 0.362^**^	Sweden	0.630^**^	Germany	− 0.037
		Panama	− 0.739^**^	India	− 0.598^**^	Chile	− 0.355^**^			Japan	− 0.039
		Philippines	− 0.619^**^	Indonesia	− 0.585^**^	Egypt	− 0.361^**^			Mongolia	0.119
		Portugal	− 0.683^**^	Kenya	− 0.480^**^	Hong Kong	− 0.359^**^			Netherland	− 0.003
		Romania	− 0.700^**^	Malaysia	− 0.504^**^	Hungary	− 0.288^**^			Norway	− 0.041
		Saudi Arabia	− 0.607^**^	New Zealand	− 0.476^**^	Ireland	− 0.246^*^			Poland	− 0.144
		Spain	− 0.723^**^	Nigeria	− 0.517^**^	Thailand	− 0.371^**^			Taiwan	0.041
				Peru	− 0.434^**^	UK	− 0.322^**^			Vietnam	− 0.139
				South Africa	− 0.416^**^						
				South Korea	− 0.418^**^						
				United States	− 0.405^**^						

* indicates *P* ≤ 0.05; ** indicates *P* ≤ 0.01

Table 2 shows the variables entered or removed in each step of the stepwise regression model based on the *t*-value of each independent variable. All independent variables were selected in the model because each contributed significantly to the predictive ability of the model. Stay at home restrictions were the most important impact factor and were selected first selected by the model due to having the highest *t*-value, followed by daily increases in COVID-19 cases, government stringency index, social gathering restrictions, public information campaigns, public event cancellations, and workplace closures. The least important was the internal movement restrictions, which received the lowest *t*-value and thus was last selected by the model.

The stepwise regression model summary and coefficient analysis were analyzed as shown in Table 2 and Table S3. All eight independent variables had a significant impact on changes in park visitor numbers, with an *R*-value is equal to 0.535. Among the eight variables, stay at home restrictions were selected first by the model and contributed significantly to a higher R-value compared to the other variables, explaining 26.1% of the variable (Table 4). The remaining factors potentially affecting changes in visits only contributed to explaining 2.5% of the variance.

**Table 4:** Impacts of Covid-19 on park visits in selected countries and regions

Countries	Variable	Model 1	Model 2	Model 3	Model 4	Model 5	Model 6
Italy	Daily increase cases	− 0.815**	− 0.483**	− 0.375**	− 0.465**	− 0.370**	− 0.277**
	Government stringency index		− 0.469**	− 0.864**	− 1.010**	-1.254**	− 1.470**
	Internal movement restriction			0.426**	0.374**	0.382**	0.221**
	stay at home restriction				0.289**	0.287**	0.149
	Public event cancellation						0.322**
	Workplace closure						0.373**
Spain	Daily increase cases	− 0.828**	− 0.622**				
	Government stringency index		− 0.398**				
South Korea	Workplace closure	0.493**	0.384**				
	Daily increase cases		− 0.251**				
United Kingdom	Internal movement restriction	− 0.387**	− 1.618**	− 0.063			
	Social gathering restriction		1.331**	1.483**	1.484**		
	Stay at home restriction			− 1.706**	− 1.770**		
Denmark	Workplace closure	0.611**					
Sweden	Daily increase cases	0.630**					
Japan	Stay at home restriction	− 0.204**					

* indicates *P* ≤ 0.05; ** indicates *P* ≤ 0.01

A coefficient analysis was conducted to find out if the independent variables were related to changes in park visits (Appendix C). Seven independent variables were related to changes in park visitor numbers, including stay at home restrictions (std. coefficient *β* =  − 0.341, *p* < 0.001), the daily increase COVID-19 cases (std. coefficient *β* = 0.025, *p* = 0.065), the government stringency index (std. coefficient *β* = − 0.645, *p* < 0.001), social gathering restrictions (std. coefficient *β* = 0.19, *p* < 0.001), public information campaigns (std. coefficient *β* = 0.13, *p* < 0.001), public event cancellations (std. coefficient *β* = 0.126, *p* < 0.001), workplace closures (std. coefficient *β* = 0.092, *p* = 0.001), and movement restrictions (std. coefficient *β* = 0.048, *p* = 0.039).

The standardized coefficient *β* values for stay at home restrictions and government stringency index were negative, meaning that these two public response variables were significantly and negatively associated with park visitor numbers. However, the remaining independent variables, including daily COVID-19 increased cases, social gathering restrictions, public information campaigns, public event cancellations, workplace closures and movement restrictions received positive standardized coefficient β values, indicating that they were positively associated with park visits. Among these independent variables, the government stringency index received the highest absolute value for the standard coefficient, followed by the stay at home restriction, whereas the daily increase in COVID-19 cases received the lowest value. In the stepwise regression model, the higher the Beta values, the greater the importance of that variable in the model, indicating that the government stringency index is the most important independent variable in the model in the global-scale analysis.

### Regional scale analysis of the impacts of COVID-19 park visits

Regional scale impacts of COVID-19 on park visits were analyzed to further understand and address the research questions at the regional level. First, 48 countries were grouped into six categories based on the correlation between changes in park visitor numbers and increases in daily COVID-19 cases. The correlation classification included significant negative correlations, significant positive correlations and no significant correlations. Countries that received significant negative correlations were grouped into a subclassification, including very strong (Pearson correlation between 0.80 –1.00), strong (0.60 –0.79), moderate (0.40–0.59) and weak (0.20 –0.39) significant negative correlation. The countries and regions contained in each group are listed in Table 3. After classifying 48 countries and regions into 6 groups, a stepwise regression analysis was performed on each group (Table S4).

The regional scale analysis revealed that the government stringency index was the most important variable with the highest *t*-value. It was the first selected in the stepwise regression model in most groups. The coefficient of government stringency index changed from negative to positive with the change of the correlation between park visitation change and daily increased cases of COVID-19 from a very strong negative correlation to a significant positive correlation. Among the eight COVID-19 related variables, social gathering restriction and public information campaign always had significant positive associations with changes in park visits, indicating that the stricter the social gathering restriction and the more widespread the public COVID-19 information campaign, the more people went to parks and the longer they stayed. Among the strong and moderate negative correlation groups, all COVID-19 responses policies received a positive coefficient, which indicates that these variables had positive associations with increased park visitation.

### National and sub-national analyses on COVID-19 impacts on park visits

**Fig. 3:**
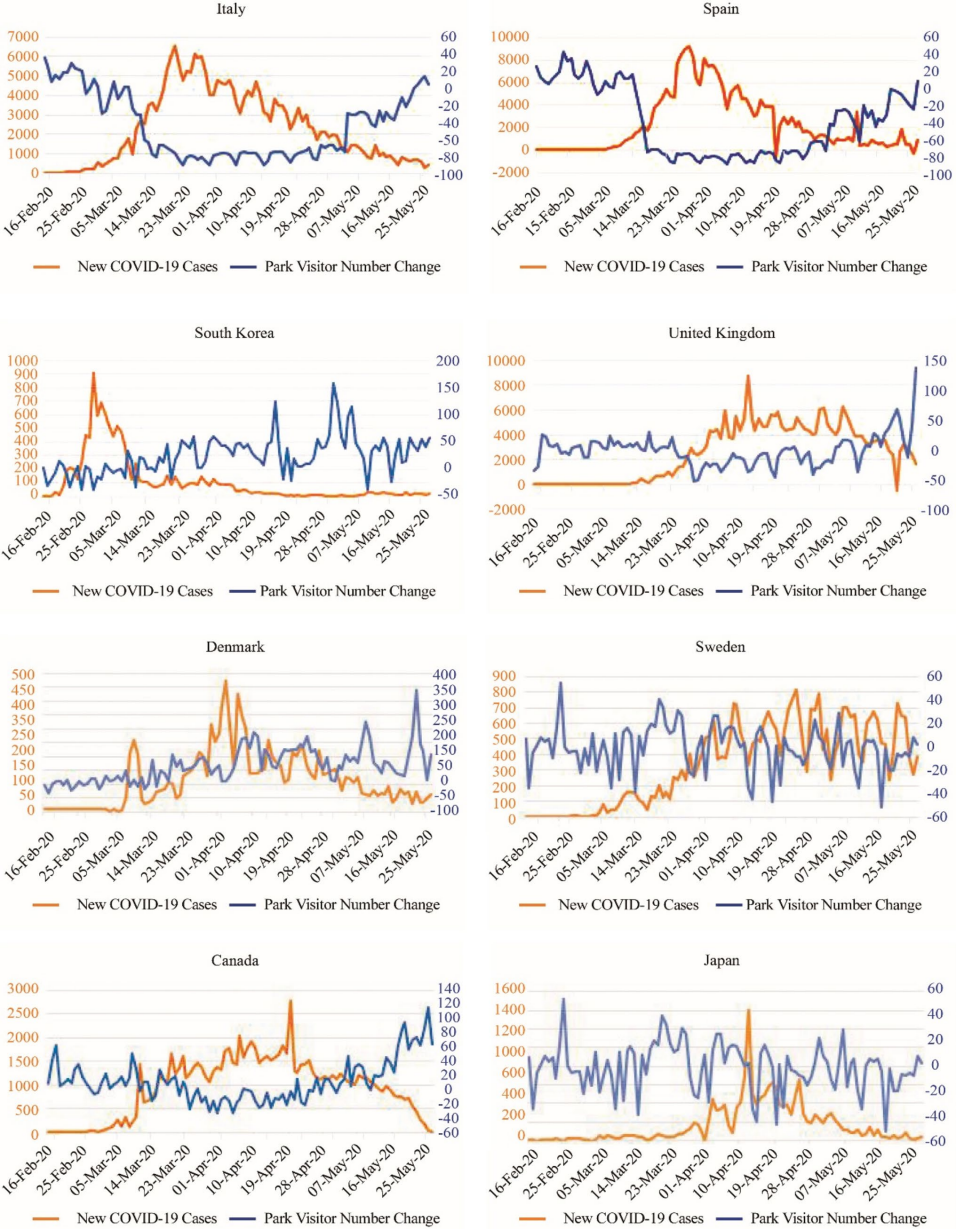
New COVID-19 cases and park visits in selected countries

Further analyses were undertaken for selected countries that represent each group. Seven countries were selected, including Italy, Spain, South Korea, United Kingdom, Denmark, Canada and Japan, representing a very strong significant negative correlation, strong significant negative correlation, moderate significant negative correlation, weak significant negative correlation, significant positive correlation and no significant correlation between park visitation change and daily COVID-19 cases, respectively. Sweden was also selected for country-specific analysis due to the unique responses adopted by the government in response to COVID-19. Impacts of COVID-19 on park visitation over time are shown in Fig. 3, and the results of each country’s stepwise regression analysis are shown in Table 4.

As of May 26 2020, the number of park visits had increased in all the selected countries, although to different degrees. The increase in park visitors in Italy, Spain, South Korea, Sweden and Japan was between 0–50% compared to the baseline, whereas the increase in park visits in the United Kingdom, Denmark and Canada was over 100%. Italy and Spain showed a similar trend in park visitor numbers, first decreasing significantly with a more than an 80% reduction during March and April 2020. After the outbreak was controlled, park visitation started to increase in May 2020, and finally ended slightly above the baseline. For South Korea, park visits first decreased during the rapid outbreak in February and the beginning of March, and then increased until reaching 150% of the baseline visitor numbers, and then eventually declining and stabilizing at 50% more than the baseline. Park visits in the United Kingdom first decreased, at the same time as new confirmed cases were increasing, and then increased rapidly after April, finally reaching 150% more than the baseline. For Denmark, park visits increased continuously during the pandemic, and visitor numbers have increased by up to 350%. Compared to Denmark, park visits in Sweden and Japan did not significantly change, whereas, in Canada, park visitor numbers increased by 80% compared to the baseline visitor numbers.

The impact factors of COVID-19 and response policies varied significantly amongst different countries. For countries with severe COVID-19 pandemics such as Italy and Spain, the COVID-19 daily case increase was the first variable selected in the stepwise regression model with the highest t-value, explaining 66.3% and 66.1% of the variance, respectively. In these countries, the daily increase in cases had a significant negative impact on the change in park visits. For countries with relatively low COVID-19 cases and deaths, such as South Korea and Denmark, most government responses were unassociated with changes in park visits. It is also worth noting that workplace closure became the most significant variable that positively affected park visitor numbers, which was first selected by the model and explained 24.3% and 37.3% of the variance.

Among the selected 10 countries, the daily increase in cases, stay at home restrictions and the government stringency index usually received negative standardized coefficients, indicating that these variables negatively affected park visits. Other responses, such as workplace closures, social gathering restrictions, public event cancellations, and internal movement restrictions tended to affect park visitation positively.

### Correlation analysis between the 17 indices and park visitation

Correlation analyses was conducted for each country using 17 indices representing economic, social and cultural dimensions. All indexes were significantly correlated with park visitation changes (Table 5). Variables that had positive impacts on park visitation included GDP/capita, life satisfaction index, environmental performance index, individualism index, personal freedom index, economic freedom index, human freedom index, environmental health index, ecosystem vitality index, life expectancy, and satisfaction with transportation. The human freedom index received the highest (positive) Pearson correlation, followed by personal freedom index, life satisfaction level, environmental performance index and GDP/capita. Negatively correlated variables included human density, power distance index, masculinity index, uncertainty avoidance index, poverty index and unemployment rate, with the power distance index having the greatest number of the highest negative value.

**Table 5:** Correlation between park visitation change and the 17 indices of economic, social and cultural factors

Variable	Pearson correlation	Sig. (2-tailed)
Human density	− 0.035^*^	0
GDP/capita	− 0.349^**^	0
Life satisfaction index	− 0.364^**^	0
Environmental performance index	− 0.359^**^	0
Power distance index	− 0.430^**^	0
Individualism index	0.338^**^	0
Masculinity index	− 0.247^**^	0
Uncertainty avoidance index	− 0.206^**^	0
Personal free index	0.369^**^	0
Economic free index	0.295^**^	0
Human free index	0.377^**^	0
Environmental health index	0.310^**^	0
Ecosystem vitality index	0.318^**^	0
Poverty index	− 0.166^**^	0
Life expectancy	0.249^**^	0
Unemployment rate	− 0.101^**^	0
Satisfaction with transportation	0.068^**^	0

* indicates *P* ≤ 0.05; ** indicates *P* ≤ 0.01, bold font indicates statistical significance

## Discussion

### Impacts of COVID-19 and associated government responses on park visitation

#### Global-scale analysis

Many governments implemented responses to COVID-19 rapidly. Near real-time information (such as the number of new cases each day) has been accompanied by dramatic changes in societal behavior (Hockings et al. 2020). At a global level, parks in most selected countries and regions have received more visitors since the COVID-19 pandemic began. The results are consistent with the findings of three regional-scale research in the United States and Germany, indicating that urban parks have received much more frequent visits during the COVID-19 pandemic (Derks et al. 2020; Fisher and Grima 2020; Rice and Pan 2020). Most government responses, including workplace closures, restrictions on social gatherings and internal movements, and public event cancellations were correlated with an increase in park visitor numbers, whereas at a global scale, the government stringency index and stay at home restrictions were correlated with reductions in park visits.

There are several possible reasons for these results, including the psychological impacts of government responses to COVID-19 and the increasing role of parks during a global pandemic. Responses such as the cancellation of public events and the restriction of social event significantly reduced people’s daily social activities. Many people went further than the governments required, altering their behavior to reduce or avoid the perceived risk, and undertaking fewer daily activities (Ferguson 2007). Previous studies have indicated that confinement and reduced social and physical contact with others during major health crises tend to cause boredom, frustration, depression and a sense of isolation from friends and family, which further distress people psychologically and physiologically (Blendon et al. 2004; Braunack-Mayer et al. 2013; Brooks et al. 2020; Cava et al. 2005; Desclaux et al. 2017; DiGiovanni et al. 2004). Because of this psychological stress and reduced social contact with others, combined with increasing concerns about mental health, people may tend to use nearby parks and green spaces to seek out connections with nature and to reduce the negative impacts caused by self-quarantine. The second possible reason for the increasing park visitation may be related to the workplace closure policy. Working at home reduces commute time, so many people have much more flexible time to spend during the quarantine. Also, work at home policies significantly change peoples’ daily routine. Based on previous research on the psychological impact of the SARS quarantine experience, a loss of routine can exacerbate peoples’ stress and frustration and increase peoples’ demand to go outdoors (Hawryluck et al. 2004; Reynolds et al. 2008; Robertson et al. 2004). If of other public places such as shopping malls and restaurants are also closed, parks become one of the only places people can go for outdoor activities (Fisher and Grima 2020), provided that they also are not closed or restricted.

#### Regional analysis

At this scale, the government stringency index was the most important variable and was the first selected by the model in most groups due to its high *t*-value. This is consistent with previous findings. Human behavior during a health crisis, such as social distancing or self-quarantine, is highly dependent on the stringency of the government response (Hussain 2020). Caley et al. (2008) found that cities and states that had a high stringency index during the Spanish Flu epidemic had a 30% to 50% lower rate of infected cases and fatality rates, respectively, compared to cities that had softer government responses.

At a regional scale, COVID-19 and the associated government responses had varied impacts on park visitation. Although the government stringency index was the first selected in the stepwise regression model in most regions, each region had different coefficients. The value of the coefficients of government stringency index changed from negative to positive with the change of the correlation between park visitation change and daily increased cases of COVID-19 from a very strong negative correlation to a significant positive correlation. This can be explained by the severity of the pandemic severity and the strictness of the government response in each region. For example, for countries that had severe cases of COVID-19 and strict government responses, the government stringency index tended to receive negative coefficient values, involving limiting the ability of people to visit parks. In countries such as Denmark and Finland, where the governments did not have strict response policies, especially at the beginning of the outbreak, and which experienced less severe cases of COVID-19, there were more positive coefficients of government stringency index and increased park visitation.

Among the selected variables, the existence of social gathering restrictions and public information campaigns always had significant positive impacts on park visitor numbers. The stricter the social gathering restriction was and the more widespread the public COVID-19 information campaign, the more people went to parks and the longer they stayed. Intensive COVID-19 information campaigns combined with the severity of the outbreak may have persuaded people to follow the rules, which they can often do when visiting a park.

#### National analyses

Park visitation in individual countries increased compared to park visitor numbers before the COVID-19 pandemic began. However, park visitation trends and levels differed between countries. The increase in park visitors in Italy, Spain, South Korea, Sweden and Japan was between 0–50% compared to the baseline, whereas the increase of park visitation in the United Kingdom, Denmark and Canada was over 100%. The differences reflect different response strategies. In countries such as Italy that experienced a severe COVID-19 outbreak and imposed very restrictive responses, park visitor numbers initially decreased significantly. Once the outbreak was controlled and the government lightened some restrictions, park visitation started to increase to levels slightly above baseline. For countries with fewer COVID-19 cases and less restrictive responses, park visitation increased continuously by over 100% compared to baseline. For example, in Canada and Denmark, the governments have “recommended stay at home” as the response policy since the start of the COVID-19 outbreak began, and more people accessed parks. It is worth noting that federal parks in Canada were actually closed to visitors, as were provincial parks in some provinces. Regional and municipal parks remained opened. Metro Vancouver (British Columbia, Canada) reported that 60% of its parks received higher visitation and utilization after the implementation of social distancing rules (Godfrey 2020).

In countries with severe COVID-19 outbreaks, the daily increase in cases was the most important variable that was negatively associated with park visitation. For countries with less severe outbreaks, neither the daily increase in cases nor government responses were associated with park visitor numbers. Workplace closures and social gathering restrictions were associated with increased visits, perhaps because people may use the time saved from their daily commute to go to parks and avoid the sense of isolation caused by reduced social contact.

In Sweden, the only variable associated with changes in visitor numbers was the daily increase in cases. This may be because Sweden sought to use herd immunity to control the disease, allowing a proportion of the population to get infected (Habib 2020; Randolph and Barreiro 2020). Bars, restaurants, and other public spaces remained open with much fewer restrictions, which may explain why only the daily increase in cases was associated with changes in visits. It may also explain why park visitation in Sweden did not significantly change during the pandemic.

### The benefits of parks and green spaces under COVID-19 Pandemic

#### Benefits of parks on mental health and stress reduction

Parks and green spaces are widely recognized as providing important public benefits, especially during health crises (National Recreation and Parks Association 2020). The spread of the disease and the implementation of government responses have resulted in some long-ignored functions of parks finally being recognized by the public (Hockings et al. 2020). Our results indicate that demand for park access increased during the pandemic. Other studies have also confirmed that during the COVID-19 pandemic, people re-evaluated the value of parks and green spaces, raising the importance of parks, especially urban parks and community parks which mostly have remained open (Fisher and Grima 2020; Freeman and Eykelbosh 2020; Nicola et al. 2020). Fisher and Grima (2020) investigated the use and value of urban and peri-urban natural areas to local communities under COVID-19 pandemic by using online survey, and 81% of respondents to a survey mentioned that the value of urban, peri-urban forests and other natural areas to them had increased or significantly increased since the pandemic began and social and work restrictions were implemented.

The COVID-19 pandemic has resulted in severe psychological burdens for many individuals around the world (Bavel et al. 2020). The impact of the pandemic, as well as self-quarantine and other response policies, on mental health is expected to be significant (Brooks et al. 2020; Freeman and Eykelbosh 2020). Drivers of negative impacts on people’s mental health include the duration of quarantine, fears of infection, frustration and boredom, and inadequate information (Brooks et al. 2020). Longer durations of self-quarantine can lead to poorer mental health, post-traumatic stress symptoms and other negative psychological impacts (Bavel et al. 2020; de Bell et al. 2020; Brooks et al. 2020; Hossain et al. 2020; Reynolds et al. 2008; Wilken et al. 2017). More specifically, based on the research done by Hawryluck et al. (2004), people with more than 10 days quarantine have shown significantly higher stress symptoms compared with those quarantined for less than 10 days. Stress associated with quarantine is compounded by fears of infection for individuals and family, loss of usual routine, restrictions on social and physical contact, and lack of clear guidelines or rationale for COVID-19 information campaigns (Brooks et al. 2020; Braunack-Mayer et al. 2013). Parks and green spaces have played a role in mitigating the psychological burdens associated with COVID-19 (Freeman and Eykelbosh 2020).

Parks can reduce stress and offer various psychological and emotional benefits during a major health crisis (Annerstedt et al. 2012; Hockings et al. 2020; Nicola et al. 2020; Seaman et al. 2010). They provide qualities like serenity, space, wildness, culture and a lush environment, which can all reduce the risk of poor mental health (Annerstedt et al. 2012). Many studies have confirmed that spending time in natural areas such as parks and green spaces can help people avoid a sense of isolation, reduce mental stress, improve sleep quality and thus reduce of the risk of depression and anxiety, and improve individuals’ resilience and manageability of life tasks (Bratman et al. 2019; Cox et al. 2017; Fong et al. 2018; Hammen 2005; Rasmussen and Laumann 2013; Roe and Aspinall, 2011).

#### Benefits of parks on physical health

Government restrictions have curtailed many daily activities but parks have compensated by providing space for physical activity spaces and fresh air. Some people have realized that a rapid and well-coordinated immune system response combined with good physical health represents the first line of defense against infection (Catanzaro et al. 2020).

The physical benefits associated with the use of parks and other natural areas have been widely recognized (Fisher and Grima 2020; Seaman et al. 2010). The Center for Disease Control and Prevention in USA has shown that park visits can improve individual and community health, resulting in a 25% increase in the perceived physical health of residents who exercise in parks at least three times per week (National Recreation and Parks Association 2020). The physical health benefits of access to urban parks is known improve cardiovascular health and pulmonary function (Lee and Lee 2014; Tamosiunas et al. 2014). Spending time in parks and other natural areas can activate Natural Killer (NK) cells in humans. NK cells are important in the human immune system as they induce virus-infected targeted cell death (Li et al. 2007; Tsao et al. 2018; Vivier et al. 2008).

#### Park benefits for reducing the risk of disease transmission and increasing social cohesion

Access to parks could reduce the risk of COVID-19 transmission and increase community and social cohesion. If parks are closed or access is otherwise limited, people may move to less desirable public spaces, such as sidewalks and pavements. However, these public spaces are not designed to encourage and maintain physical distancing (Barkhorn 2020). Parks allow people to spread out, reducing crowding in less desirable areas (Freeman and Eykelbosh 2020; Public Health England 2014).

Parks, particularly community parks, may improve social cohesion at a community level and help build a sense of integration and inclusion amongst residents in their communities. Increased social and community cohesion and feelings of integration and belonging can reduce the risk of antisocial behavior especially during public health crises (Seaman et al. 2010).

### Recommendations and future park planning and development

#### Utilization of parks during the COVID-19 pandemic

We suggest that the use of community parks and urban parks should not be restricted during the COVID-19 outbreak. Parks provide important ecosystem services that mitigate some of the stress associated with COVID-19 and ensure the mental and physical health of individuals. The implementation of government responses to COVID-19 significantly restricts peoples’ social and physical contact with others. Many parks provide an opportunity for people to be outside without violating social distancing restrictions (Eagles 2020), and in addition to provide benefits that help people cope with physical and mental challenges posed by the pandemic. It is also worth noting that urban residents in countries with high infection rates and confirmed cases should also be encouraged to go to nearby community parks and green spaces as long as they follow the protective guidelines and instructions such as maintaining physical distancing from each other. Take the United States as an example, the government conducted shelter-in-place orders since March 2020, which has significantly restricted residents’ movements and human interaction (Gostin and Wiley 2020). Under the closure of workplaces, schools, shopping malls, restaurants and other public places, the access of parks and open green spaces in the United States also got restricted by the policies. However, studies have been confirmed that these restrictions on parks and green spaces could possibly cause people severe physical and mental health issues especially those living in urban areas (Sallis and Pratt 2020; Slater et al. 2020).

With development of the COVID-19 pandemic, people have gradually increased their awareness to protect themselves against COVID-19 by understanding and practicing social-distancing rules in this new normal. Combined with the overall global pandemics have been brought relatively under control with more understanding to manage confirmed cases and reduce potential spread, some government restrictive policies such as stay at home restrictions are gradually liberalized in the community, allowing people to access their nearby parks and greenspaces to gain larger spaces for some recreational activities with more self-protections. On the basis that nowadays people understand and strictly abide by the social-distancing rules under the loosening of stay-at-home restrictions, parks and green spaces may be utilized as important natural services to personal well-being and the society. The ecological services and functions provided by parks and greenspaces allow people to access and conduct physical exercise, reduce the mental stress while maintaining physical distance under the COVID-19 pandemic.

#### Park visitation and associated economic, cultural and social index

We also analyzed the correlation between park visitation and selected economic, cultural and social indexes for each country and region. Access to parks was unequal, and was significantly correlated with economic development, environmental performance and cultural characteristics.

The economic development index includes GDP per capita, power distance index, economic freedom index, poverty index and unemployment rate. Results indicate that park visitation was greater in more developed economies. Poor economic performance could lead to less access to parks, and thus compound the more negative impacts of COVID-19. This is consistent with Larson et al. (2016), Laster Pirtle (2020) and Nesbitt et al. (2019), who found unequal access to urban green spaces in US cities, with access being negatively correlated with poverty rate. The correlation analysis indicated that all the environmental performance factors had significant positive associations with park visitation.

Population density was significantly negatively associated with park visitation. Research done in Berlin showed that congested city streets and crowded apartment blocks may have a higher risk of infection than being in open spaces such as parks and green spaces, and raise the risk of mental health issues (Saunders and Evans 2020). The provision of urban parks and community greenspaces in areas with high population density appears to be particularly important.

## Conclusion

In this study, we analyzed the association between the COVID-19 pandemic and associated government responses and park visitation at global, regional and national scales. Park visitation increased compared with a baseline developed before the outbreak began. Public policies that restricted people’s social and physical contacts were associated with increased park visitation. Our findings suggest that the demand for access to parks and outdoor green spaces increased during the pandemic.

The study has a number of limitations. According to Google Community Mobility Report, the park visitation change was calculated compared with the “baseline” for the corresponding day of the week. The baseline visitor number was determined by the median value of park visitors from January 3rd to February 6th, 2020, which is immediately before COVID-19 started to become widespread. However, the change could also be related to the seasonal change of the weather and climate. Therefore, future work is needed to eliminate the seasonal effects on park visitation change. Future research also could focus on (1) further exploring the post-COVID-19 park visitor numbers and people’s opinions about parks, and (2) conducting a community-scale correlation and stepwise regression model, as well as an assessment of the equality of access to parks amongst local residents.

## Electronic supplementary material

Below is the link to the electronic supplementary material.

Supplementary file1 (DOCX 29 kb)
