# Supplementary material for: Impacts of COVID-19 pandemic on urban park visitation: a global analysis
Source: J For Res (Harbin). 2020 Nov 12;32(2):553–67. doi: 10.1007/s11676-020-01249-w (PMC7660132; doi:10.1007/s11676-020-01249-w)
Supplement: Supplementary file 1 — Supplementary file1 (DOCX 29 kb) [file 11676_2020_1249_MOESM1_ESM.docx]

**Table S1** Different Levels of Each COVID-19 Response Policy

| Response policy | Categories | | | | |
| --- | --- | --- | --- | --- | --- |
| Restrictions on stay at home | No measures | Recommended restrictions | Required (except essentials) | Required (few exceptions) |  |
| Cancellation of public events | No measures | Recommended cancellations | Required cancellations |  |  |
| Restrictions on public gatherings | No measures | >1000 people | 100-1000 people | 10-100 people | <10 people |
| Public information campaigns | No measures | Public officials urging caution | Coordinated information campaign | |  |
| Restrictions on internal movement | No measures | Recommend restriction | Restrict movement |  |  |
| Workplaces closures policy | No measures | Recommended | Required for some | Required for all but key workers |  |
| Government Stringency Index | a value between 0 and 100 | | | | |

**Table S2** Descriptive information and data source for each index

| Index | Description | Data Source |
| --- | --- | --- |
| Population density | People per sq.km of land area | [The World Bank Group (2020)](#bookmark=id.f7q90joqzbx3) |
| GDP/capita | Country's economic output that accounts for its number of people | [International Monetary Fund (2020)](#bookmark=id.ulqbtmqv88kx) |
| Life satisfaction index | Cantril Ladder (0=worst; 10=best) | [Helliwell et al. (2019)](#bookmark=id.mmbh8v2bh64j) |
| Environmental performance index | State of environmental sustainability of each country | [Yale University (2020)](#bookmark=id.y2x70c653fhm) |
| Individualism index | The level to which individuals are integrated into groups | [Hofstede Insight (2020a)](#bookmark=id.ps64tljeu8s1) |
| Masculinity index | The distribution of roles between the genders | [Hofstede Insight (2020b)](#bookmark=id.p0w0tc33d5mo) |
| Power distance index | Distribution of power and wealth between people of each country | [Hofstede Insight (2020c)](#bookmark=id.4y0evfaoiafc) |
| Uncertainty avoidance index | A society’s tolerance for uncertainty and ambiguity | [Hofstede Insight (2020d)](#bookmark=id.7rj1ywal3xzn) |
| Personal freedom index | The freedom of an individual to have opinion and expression | [Vásquez & Porčnik (2018](#bookmark=id.gubq8f9gdpud)) |
| Economic freedom index | Country’s trade freedom, business freedom, investment freedom, and property rights. | [Vásquez & Porčnik (2018)](#bookmark=id.gubq8f9gdpud) |
| Human freedom index | A comprehensive measure of the state of human freedom, including civil, personal and economic aspects | [Vásquez & Porčnik (2018)](#bookmark=id.gubq8f9gdpud) |
| Environmental health index | Includes air quality, water and sanitation, and heavy metal condition | [Yale University (2018)](#Yale2018) |
| Ecosystem vitality index | Includes biodiversity and habitat, forests, fisheries, climate and energy, pollution, water resources and agriculture | [Yale University (2018)](#Yale2018) |
| Poverty index | An indication of the poverty of the community in a country | [Our World in Data (2020)](#our) |
| Life expectancy | A statistical measure of the average time an individual is expected to live | [Our World in Data (2020)](#our) |
| Unemployment rate | The number of unemployed persons divided by the number of persons in the labour force | [Our World in Data (2020)](#our) |
| Public transport satisfaction | People’s satisfaction level on public transportation | [Our World in Data (2020](#our)) |

**Table S3** Coefficients analysis in the stepwise regression model for the impact Covid-19 and government responses on park visitor numbers

|  |  |  |  |  |  |  | 95.0% Confidence Interval for B | |  |  |
| --- | --- | --- | --- | --- | --- | --- | --- | --- | --- | --- |
| Model |  | Unstandardized B | Coefficients Std.Error | Standardized Coefficients | t | sig. | Lower Bound | Upper Bound | Tolerance | VIF |
| 1 | (Constant) | 7.911 | 0.777 |  | 10.184 | **0.000** | 6.388 | 9.433 |  |  |
|  | Stay at home restriction | -21.787 | 0.526 | -0.511 | -41.387 | **0.000** | -22.819 | -20.755 | 1 | 1 |
| 2 | (Constant) | 7.863 | 0.775 |  | 10.152 | **0.000** | 6.345 | 9.382 |  |  |
|  | Stay at home restriction | -22.455 | 0.539 | -0.527 | -41.641 | **0.000** | -23.512 | -21.398 | 0.948 | 1.055 |
|  | Daily increase cases | 0.001 | 0 | 0.068 | 5.405 | **0.000** | 0.001 | 0.001 | 0.948 | 1.055 |
| 3 | (Constant) | 12.295 | 1.228 |  | 10.011 | **0.000** | 9.888 | 14.703 |  |  |
|  | Stay at home restriction | -18.698 | 0.972 | -0.439 | -19.246 | **0.000** | -20.603 | -16.793 | 0.291 | 3.44 |
|  | Daily increase cases | 0.001 | 0 | 0.065 | 5.164 | **0.000** | 0.001 | 0.001 | 0.945 | 1.058 |
|  | Government stringency index | -0.145 | 0.031 | -0.104 | -4.644 | **0.000** | -0.207 | -0.084 | 0.299 | 3.35 |
| 4 | (Constant) | 14.377 | 1.265 |  | 11.363 | **0.000** | 11.896 | 16.857 |  |  |
|  | Stay at home restriction | -18.557 | 0.968 | -0.435 | -19.175 | **0.000** | -20.454 | -16.66 | 0.291 | 3.442 |
|  | Daily increase cases | 0.001 | 0 | 0.051 | 3.957 | **0.000** | 0 | 0.001 | 0.915 | 1.093 |
|  | Government stringency index | -0.319 | 0.041 | -0.229 | -7.733 | **0.000** | -0.4 | -0.238 | 0.17 | 5.875 |
|  | Social gathering cancelation | 3.606 | 0.561 | 0.148 | 6.425 | **0.000** | 2.505 | 4.706 | 0.282 | 3.544 |
| 5 | (Constant) | 4.387 | 1.892 |  | 2.319 | **0.020** | 0.679 | 8.096 |  |  |
|  | Stay at home restriction | -16.424 | 1.009 | -0.385 | -16.277 | **0.000** | -18.402 | -14.446 | 0.265 | 3.78 |
|  | Daily increase cases | 0.001 | 0 | 0.044 | 3.441 | **0.001** | 0 | 0.001 | 0.91 | 1.099 |
|  | Government stringency index | -0.494 | 0.048 | -0.355 | -10.307 | **0.000** | -0.588 | -0.4 | 0.125 | 8 |
|  | Social gathering cancelation | 4.298 | 0.567 | 0.176 | 7.581 | **0.000** | 3.186 | 5.409 | 0.274 | 3.652 |
|  | Public information campaign | 8.934 | 1.263 | 0.11 | 7.074 | **0.000** | 6.458 | 11.41 | 0.615 | 1.626 |
| 6 | (Constant) | 4.301 | 1.889 |  | 2.277 | **0.023** | 0.597 | 8.004 |  |  |
|  | Stay at home restriction | -14.879 | 1.081 | -0.349 | -13.762 | **0.000** | -16.998 | -12.759 | 0.23 | 4.352 |
|  | Daily increase cases | 0 | 0 | 0.038 | 2.936 | **0.003** | 0 | 0.001 | 0.896 | 1.116 |
|  | Government stringency index | -0.679 | 0.067 | -0.487 | -10.136 | **0.000** | -0.81 | -0.548 | 0.064 | 15.654 |
|  | Social gathering cancelation | 4.507 | 0.569 | 0.185 | 7.928 | **0.000** | 3.393 | 5.622 | 0.271 | 3.684 |
|  | Public information campaign | 9.226 | 1.263 | 0.113 | 7.304 | **0.000** | 6.75 | 11.703 | 0.613 | 1.631 |
|  | Public event cancelation | 5.557 | 1.41 | 0.107 | 3.942 | **0.000** | 2.794 | 8.321 | 0.199 | 5.013 |
| 7 | (Constant) | 4.534 | 1.889 |  | 2.400 | **0.016** | 0.831 | 8.238 |  |  |
|  | Stay at home restriction | -14.651 | 1.083 | -0.344 | -13.531 | **0.000** | -16.774 | -12.529 | 0.229 | 4.373 |
|  | Daily increase cases | 0 | 0 | 0.03 | 2.278 | **0.023** | 0 | 0.001 | 0.861 | 1.161 |
|  | Government stringency index | -0.809 | 0.079 | -0.581 | -10.186 | **0.000** | -0.965 | -0.654 | 0.045 | 22.07 |
|  | Social gathering cancelation | 4.522 | 0.568 | 0.186 | 7.961 | **0.000** | 3.409 | 5.636 | 0.271 | 3.685 |
|  | Public information campaign | 10.049 | 1.291 | 0.124 | 7.786 | **0.000** | 7.519 | 12.58 | 0.586 | 1.706 |
|  | Public event cancelation | 6.017 | 1.416 | 0.116 | 4.248 | **0.000** | 3.24 | 8.793 | 0.197 | 5.07 |
|  | Workplace closure | 3.063 | 1.006 | 0.086 | 3.046 | **0.002** | 1.091 | 5.034 | 0.186 | 5.385 |
| 8 | (Constant) | 4.762 | 1.892 |  | 2.518 | **0.012** | 1.054 | 8.47 |  |  |
|  | Stay at home restriction | -14.52 | 1.084 | -0.341 | -13.392 | **0.000** | -16.646 | -12.395 | 0.228 | 4.388 |
|  | Daily increase cases | 0.000 | 0.000 | 0.025 | 1.845 | 0.065 | 0 | 0.001 | 0.83 | 1.205 |
|  | Government stringency index | -0.898 | 0.09 | -0.645 | -9.946 | **0.000** | -1.075 | -0.721 | 0.035 | 28.504 |
|  | Social gathering cancelation | 4.636 | 0.571 | 0.19 | 8.126 | **0.000** | 3.518 | 5.755 | 0.269 | 3.719 |
|  | Public information campaign | 10.547 | 1.313 | 0.13 | 8.035 | **0.000** | 7.973 | 13.12 | 0.566 | 1.766 |
|  | Public event cancelation | 6.528 | 1.437 | 0.126 | 4.541 | **0.000** | 3.71 | 9.346 | 0.191 | 5.225 |
|  | Workplace closure | 3.271 | 1.01 | 0.092 | 3.238 | **0.001** | 1.29 | 5.252 | 0.184 | 5.439 |
|  | Movement restrictions | 2.276 | 1.103 | 0.048 | 2.064 | **0.039** | 0.114 | 4.438 | 0.268 | 3.726 |
|  | Dependent Variable: park visitor number change | | |  | | | | | | |

(Note:bold font indicates statistical significance, * indicates *P* ≤ 0.05; ** indicates *P* ≤ 0.01, bold font indicates statistical significance.)

**Table S4** Stepwise regression analysis of COVID-19 impacts on park visitation in group level

| Variable | Model 1 | Model 2 | Model 3 | Model 4 | Model 5 | Model 6 | Model 7 | Model 8 | Model 9 |
| --- | --- | --- | --- | --- | --- | --- | --- | --- | --- |
| Very strong significant negative correlation group *(Italy, Singapore)* | | | | | | | | | |
| Government stringency index | -0.817** | -1.641** | -2.011** | -2.392** | -2.574** |  |  |  |  |
| Internal movement restriction |  | 0.892** | 1.064** | 0.809** | 0.902** |  |  |  |  |
| Public event cancellation |  |  | 0.299** | 0.381** | 0.330** |  |  |  |  |
| Workplace closure |  |  |  | 0.601** | 0.574** |  |  |  |  |
| Social gathering restriction |  |  |  |  | 0.186** |  |  |  |  |
| Strong significant negative correlation group  *(Austria, France, Mexico, Panama, Philippines, Portugal, Romania, Saudi Arabia, Spain)* | | | | | | | | | |
| Government stringency index | -0.785** | -0.713** | -0.426** | -0.791** | -1.083** | -1.371** | -1.851** | -1.954** | -1.836** |
| Daily increase cases |  | -0.231** | -0.207** | -0.202** | -0.205** | -0.186** | -0.194** | -0.198** | -0.193** |
| Stay at home restriction |  |  | -0.329** | -0.303** | -0.200** | -0.199** | -0.059 |  |  |
| Social gathering restriction |  |  |  | 0.365** | 0.472** | -0.559** | 0.560** | 0.569** | 0.580** |
| Public information campaign |  |  |  |  | 0.183** | 0.207** | 0.234** | 0.244** | 0,235** |
| Internal movement restriction |  |  |  |  |  | 0.212** | 0.260** | 0.265** | 0.252** |
| Public event cancellation |  |  |  |  |  |  | 0.320** | 0.355** | 0.345** |
| Workplace closure |  |  |  |  |  |  |  |  | -0.112 |
| Moderate significant negative correlation group  *(Argentina, Belgium, Columbia, India, Indonesia, Kenya, Malaysia, New Zealand, Nigeria, Peru, South Africa, South Korea, United States)* | | | | | | | | | |
| Government stringency index | -0.629** | -0.653** | -0.898** | -1.121** | -1.159** | -1.458** |  |  |  |
| Daily increase cases |  | 0.198** | 0.181** | 0.144** | 0.142** | 0.118** |  |  |  |
| Public event cancellation |  |  | 0.279** | 0.303** | 0.303** | 0.399** |  |  |  |
| Workplace closure |  |  |  | 0.235** | 0.245** | 0.261** |  |  |  |
| Public information campaign |  |  |  |  | 0.063** | 0.095** |  |  |  |
| stay at home restriction |  |  |  |  |  | 0.211** |  |  |  |
| Weak significant negative correlation group  *(Australia, Bolivia, Brazil, Chile, Egypt, Hong Kong, Hungary, Ireland, Thailand, UK)* | | | | | | | | | |
| Workplace closure | -0.592** | -0.356** | -0.423** | -0.306** | -0.269** |  |  |  |  |
| Internal movement restriction |  | -0.310** | -0.324** | -0.225** | -0.201** |  |  |  |  |
| Public event cancellation |  |  | 0.096 | 0.264** | 0.257** |  |  |  |  |
| Government stringency index |  |  |  | -0.360** | -0.461** |  |  |  |  |
| Public information campaign |  |  |  |  | 0.076 |  |  |  |  |
| Significant positive correlation group (*Denmark, Finland, Sweden*) | | | | | | | | | |
| Government stringency index | 0.569** | 0.530** | 0.252 | 0.145 |  |  |  |  |  |
| Daily increase cases |  | 0.166** | 0.203** | 0.225** | 0.246** |  |  |  |  |
| Workplace closure |  |  | 0.290 | 0.585** | 0.737** |  |  |  |  |
| Public event cancellation |  |  |  | -0.240** | -0.263** |  |  |  |  |
| No significant correlation group  *(Canada, Ecuador, Germany, Japan, Mongolia, Netherlands, Norway, Poland, Taiwan, Vietnam)* | | | | | | | | | |
| Internal movement restrictions | -0.194** | -0.353** | -0.091 | -0.075 | -0.043 |  |  |  |  |
| Social gathering restriction |  | 0.291** | 0.597** | 0.604** | 0.656** | 0.669** | 0.647** |  |  |
| Government stringency index |  |  | -0.554** | -0.991** | -1.089** | -1.137** | -1.136** |  |  |
| Public event cancellation |  |  |  | 0.478** | 0.445** | 0.445** | 0.446** |  |  |
| Public information campaign |  |  |  |  | 0.112** | 0.119** | 0.118** |  |  |
| Daily increase cases |  |  |  |  |  |  | 0.060* |  |  |

(Note: * indicates *P* ≤ 0.05; ** indicates *P* ≤ 0.01, bold font indicates statistical significance.)
